# Miscellany

**Published:** 1894-10

**Authors:** 


### Miscellany.

Collection and Preservation of Anatomical Material.—The following circular, which is self-explanatory, has been issued:

At the last meeting of the Association of American Anatomists, the undersigned committee was appointed to "consider the question of the collection and preservation of anatomical material, and to report, at the next meeting, what in their opinion are the best means of accomplishing these objects.

In order to make the work of the committee as comprehensive as possible, and to obtain information which will be of service in arriving at definite conclusions as to the best methods of accomplishing the purposes indicated in the resolution, the committee has deemed it desirable to send to the teachers of anatomy, not only in this country, but abroad, this circular letter, with the questions appended, and respectfully to request answers thereto, as fully as they can be made.

1. Is anatomical material obtained in accordance with a legal enactment, wholly or in part ?

2. If there is an anatomical law in your country or state, please send a copy of it to the chairman of this committee, Dr. J. Ewing Mears, 1429 Walnut street, Philadelphia, Pennsylvania. Please state whether the law is satisfactory, whether it is readily obeyed by those upon whom duties are imposed by it, and mention any improvements you would suggest as to its requirements.

3. Is the material received in good condition ?

4. What disposal is ultimately made of the remains ?

5. Please state what means are employed to preserve anatomical material for the purposes of dissection or operative surgery. If injections of preservative fluids are used, state their composition and the methods of use, at what point injections are made, whether at the heart or in the large arteries, and their effect in accomplishing the preservation, with any changes in the color or character of the tissues. What length of time can material be used in dissection by the methods employed by you ? If preservation by means of "cold storage" is employed, please state the cost of the machinery which it was necessary to construct for this purpose, and what means are taken to prevent decomposition after the subject is placed upon the table for dissection.

6. Please state the cost, by the method employed by you, for each subject, (*a*) for receiving it, (*b*) for injecting and preserving it.

7. Do you obtain an adequate supply of material for the purposes of anatomical instruction ? How many students are assigned to each subject, and what is the method of allotment ?

8. Please give any information which you may deem of importance. As the report will be general in character, the name of the informant or institution will not be mentioned by the committee unless requested.

Your compliance with the request of the committee, *at an early date*, will be fully appreciated as rendering assistance to it in accomplishing its work, and it desires to thank you for the same in advance.

J. EWING MEARS, M. D.,
THOMAS DWIGHT, M. D.,
JOSEPH D. BRYANT, M. D.,

1429 WALNUT STREET, Philadelphia, Pa. *Committee.*

PHRENOLOGY A HUMBUG.—Physiologists looked askance at phrenology from the beginning. It is not disputed that the brain is the seat of the intellect, the will and the emotions, and that their manifestation in any given subject is determined by the volume, conformation and texture of the brain substance ; but the method of Gall and Spurzheim, which assigns to every human characteristic its specific locality, has been generally rejected ; and now comes a writer in *The Pall Mall Magazine*, who pronounces it a "humbug" and pokes fun at it. He says :

"There is something fascinating in the thought of those precise bumps represented upon phrenological charts, each one a chamber for some busy occupant—some genius of color or song, some tiny cupid of love, some tireless calculator, some gentle sprite of benevolence or parental affection. It is so interesting to imagine all knowledge exactly filed and docketed and pigeonholed, like precious papers stored in the sections of an antique cabinet full of ingenious subdivisions and hidden springs and secret doors.

"We, too, in our youthful days, were fired with enthusiasm for this bewitching science, and read phrenological books, and studied plaster casts, and marveled at many a speculative flight. But, to our dismay, at the first touch of precise investigation the soaring fabric tumbled to the earth.

"Phrenology places the perceptive organs immediately above the eyebrows, and points to the frontal projections, so marked in many heads, as indications of development. It is, however, painfully disheartening to the phrenological student to discover, as he may do by dividing a skull, that there is no brain, but only mucus, against these suggestive bumps, and that the brain lies more than half an inch back, behind a second and inner formation of bone. So much for the 'perceptive bumps.'

"In the second place, phrenology takes cognizance only of the top, front, rear and sides of the head, but wholly ignores the organs which rest upon the base of the cranium. Let the reader imagine a skull severed by a horizontal circular line drawn from the brow, above the opening of the ears, to the back of the head : if the upper portion be removed, it will be seen that in the cup thus exposed, above the roof of the mouth, are some of the largest and most important of all the 'bumps.' Phrenology, being unable to reach these (excepting after death), simply passes them by in silence, which is often an easy way of getting over an otherwise insuperable difficulty.

"We do not dispute the fact that there are subdivisions of the brain, nor that, within recent years, the precise position of two or three mental organs has been determined. But these discoveries have been made not only without the aid of the bumps, but in contradiction of the whole theory of phrenology. Probably the general conformation of a head indicates somewhat of the nature of the brain within, just as a physiognomist reads much of a man's character by his expression. The awkward facts remain, however, that men of the highest order of intelligence sometimes have their phrenological bumps all wrong, and that physiognomy will fail to detect that yonder meditative youth, with the calm, intellectual brow, and poetic eyes and benevolent expression, is none other than 'Jim the penman.' Were phrenology an exact science, and were the 'bumps' an unfailing guide, any one might qualify him or herself to become a phrenologist; this, however, its professors admit, is not the case. They claim for themselves peculiar and exceptional aptitudes, which common people, and even mere men of science, do not possess. It must, indeed, be hard to say, as the phrenologist is constantly called upon to do, whether an apparent bump is actually a projection, or whether it only seems to be so from the depression of adjacent organs.

"The phrenologist resembles the celebrated character who attempted to judge of the contents of a wine-cellar by sniffing at its keyhole. He has been likened, by Oliver Wendell Holmes, to one who, fumbling about the outside of a locked iron safe, should assume to say what is within. 'Beneath this point,' cries the 'professor,' touching a particular spot on the polished surface, 'lies a bag of gold, to the right rests a bundle of musty deeds, and here my fingers tingle over a jewel-casket.' Yet who knows but that the safe may be as empty as the science of phrenology itself?"
—*Literary Digest.*

---

The Carpenter Milk Company, at 384 Massachusetts avenue, Buffalo, has, at the suggestion of a number of the prominent members of the medical profession, put in a well-equipped plant for the production of sterilized milk. The value of sterilized milk as a diet during the prevalence of Summer diarrheas, and to prevent fermentation in the digestive tract, is well known, and it is believed to be of importance in preventing the spread of tubercular diseases.

Physicians can prescribe sterilized milk from the Carpenter Company's establishment with a certainty that the sterilization is thorough, as tests by different experts have repeatedly demonstrated.

---

Numerical Strength of the Different Schools of Medicine in the United States.—Dr. Scudder, of Cincinnati, has prepared a carefully revised list of the number of practitioners of each school of medicine in the United States. He estimates that the total number of physicians of all schools, according to a recent list issued by the Gardiner Company, of New York, is 118,453. After making allowances for duplicates and dead addresses, Polk estimates the entire number at 106,633. Of these, 72,028 are estimated as regular physicians, 9,648 homeopathic, 10,292 as eclectic, and 1,553 as physio-medical; the remaining 11,524 as unclassified, many of these probably being duplicates, dead addresses and patent medicine men.

The number of homeopathic physicians in the United States has been variously estimated at from 10,000 to 18,000. The above estimate (9,648) is not far from the right figure.

The eclectics lead them with 10,292. There are more eclectics than homeopaths in thirty states and territories out of fifty. The states in which they lead very materially are Arkansas, Georgia, Indiana, Indian Territory, Kentucky, Missouri, Ohio, Oklahoma, Tennessee, Texas and West Virginia.

The number of homeopaths exceeds that of the eclectics in nineteen states and territories. Their chief strength lies in the old Eastern states, like Maine, Massachusetts, New Jersey, New York, Pennsylvania, Rhode Island and Vermont.

The chief strength of the physio-medical school lies in Indiana, Illinois and Ohio.— *Cleveland Medical Gazette.*

---

"When in Doubt, Operate."—The silly and ignorant crusade against what people are pleased to call vivisection seems to be joining hands with an even more silly and ignorant attack on life-saving surgery. To show the depths to which it is possible to descend, the old aphorism, "when in doubt, operate," has been twisted from its meaning and paraded before the public as an illustration of the awful hankering after operation which is supposed to permeate and degrade the surgical mind. It would be difficult to imagine a greater perversion of truth than is conveyed in the suggestion that, in such a case of doubt as is there meant, the operation would be undertaken for the surgeon's instruction rather than for the patient's good. In case of hernia there can be no more life-saving rule than this of operating if there is any doubt as to whether it may not be strangulated. It was an aphorism derived from experience long before operations had the safety now given them by antiseptics; how much more should it hold true now? To give the patient "the benefit of the doubt" means to give him the benefit of the operation, and none but those who know nothing of the subject could have founded on the phase an argument against the tendencies of modern surgery.—*Medical Fortnightly.*

VIRCHOW'S DAY'S WORK.—Prof. Rudolph Virchow, although he will be seventy-three years old on the thirteenth of October, when recently asked at what time he was accustomed to retire, replied: "When my day's work is done. It may be one o'clock, or three or five in the morning, but it is my rule not to sleep until I have finished what I have to do."

That this plan of working has not entirely broken him down was demonstrated by his recent attendance on the Congress of Americanists at Stockholm, and returning home, reaching Berlin in the morning after two days and three nights of continuous travel, he delivered an address before a scientific association, and at five o'clock the same afternoon, took his departure for an equally long and continuous journey to Serajevo, the capitol of Bosnia, to attend an archeological and anthropological congress, at which he presided.—*Philadelphia Polyclinic.*

---

DANGER IN COLORED PAPER.—Some of the colored paper supplied for kindergarten work is said to have been proved by analysis to contain a small quantity of arsenic, writes Miss Elizabeth R. Scovil in the October *Ladies' Home Journal.* The only safeguard is to purchase it from a reliable dealer. The large firms importing or manufacturing supplies for use in kindergartens have an interest in furnishing the best. One firm has long avoided the use of paper containing poison, preferring that which is absolutely safe, even though the colors may not be as bright.

---

THE Charles Roome Parmele Company, manufacturing chemists, 98 William street, New York, has been organized and announced as successors to E. M. Johnson Company in the manufacture and sale of the following preparations as specialities: "Arsenauro" (Liquor auri et arsenii bromidi); "Mercauro" (Liquor auri arsenii hydrargyri bromidi); "Calcauro" (Liquor auri arsenii et calcii bromidi); "Manganauro" (Liquor auri arsenii et mangani bromidi).

---

THE Emperor of Austria has granted to the widow of the late Professor Billroth, a yearly pension of 2,000 florins. This is to be interpreted as a mark of special favor, because according to the law of Austria the pension allowed to widows of professors is only 600 florins. As the distinguished surgeon is understood to have left little or no private fortune, the Emperor's grateful act has given general satisfaction.—*Chicago Medical Recorder.*

Dr. G. Frank Lydston will, on October 1st, begin a second course of illustrated lectures in Regional Surgery at the Masonic Hospital, of Chicago. These lectures will be given every Monday at 8 p. m.—*Chicago Medical Recorder.*

---

Buffalo University Medical College began its forty-ninth annual session Monday evening, September 4, 1894. The opening address was delivered by Prof. F. W. Hinkel, M. D., who discoursed upon the relations of the State to higher medical education. The influence of such an address in teaching students to respect medical practice laws must necessarily be good. We hope to publish the able address in full in our November issue.

---

Legality of Vaccination.—The Supreme Courts of the States of New York and Pennsylvania have definitely decided that children who have not been vaccinated can be prevented from attending public schools.

This is as it should be, and the sooner other states take cognizance of these decisions and enforce compulsory vaccination laws, the quicker will there be a great advance toward permanently stamping out small-pox.—*Medical Age.*

---

A good plan is suggested by the North Carolina Board of Medical Examiners, viz.: When complaints are made by candidates who fail to pass successful examinations, that the questions submitted to the applicant, together with his written answers, be published.—*Charlotte Medical Journal.*

---

St. Louis has an ambulance whose motive power is electricity from the street car lines. From the description, it must run on the tracks, so there is possibly a switch to the hospital. *Life,* long since, had a cartoon showing an ambulance attached to a Broadway cable car, in which operations were in progress as the cars moved. The Baltimore car lines furnish material for such an amphitheatre on wheels.—*Maryland Medical Journal.*

